# Supplementary material for: Alveolar proteins stabilize cortical microtubules in Toxoplasma gondii
Source: Nat Commun. 2019 Jan 23;10:401. doi: 10.1038/s41467-019-08318-7 (PMC6344517; doi:10.1038/s41467-019-08318-7)
Supplement: Supplementary file 1 — Supplmentary Information [file 41467_2019_8318_MOESM1_ESM.pdf]

Alveolar proteins stabilize cortical microtubules in *Toxoplasma gondii*

Clare R. Harding[1,*], Matthew Gow[2], Joon Ho Kang[3,4], Emily Shortt[1], Scott R. Manalis[4,5,6], Markus Meissner[2,7], and Sebastian Lourido[1,8*]

[1]Whitehead Institute for Biomedical Research, Cambridge, MA, USA.
[2]Wellcome Centre for Molecular Parasitology, Institute of Infection, Immunity & Inflammation, University of Glasgow, Glasgow, UK.
[3]Department of Physics, Massachusetts Institute of Technology, Cambridge, MA, USA.
[4]Koch Institute for Integrative Cancer Research, Massachusetts Institute of Technology, Cambridge, MA, USA.
[5]Department of Biological Engineering, Massachusetts Institute of Technology, Cambridge, MA, USA.
[6]Department of Mechanical Engineering, Massachusetts Institute of Technology, Cambridge, MA, USA.
[7]Department of Veterinary Sciences, Ludwig-Maximilians-Universität, Munich, Germany.
[8]Biology Department, Massachusetts Institute of Technology, Cambridge, MA, USA.
[*]To whom correspondence should be addressed: harding@wi.mit.edu, lourido@wi.mit.edu.

SUPPLEMENTARY INFORMATION

SUPPLEMENTARY FIGURES 2

Supplementary Figure 1. GAPM foci are commonly seen at alveoli sutures. 2

Supplementary Figure 2. Conditional depletion of GAPM2a results in a block in the lifecycle and loss of cortical microtubules.......... 3

Supplementary Figure 3. Quantification of GAPM1a protein based on tagged virion standard 4

Supplementary Figure 4. Depletion of GAPM1a-AID results in a decrease in parasite length and increase in circularity. 5

Supplementary Figure 5. Depolymerisation of microtubules does not affect GAPM1a localization. ... 6

SUPPLEMENTARY TABLES.......... 7

Supplementary Table 1. Number of cortical microtubules and surface area across the apicomplexan zoites. 7

Supplementary Table 2. List of primers used in this study.......... 9

SUPLEMENTARY REFERENCES 10

SUPPLEMENTARY FIGURES

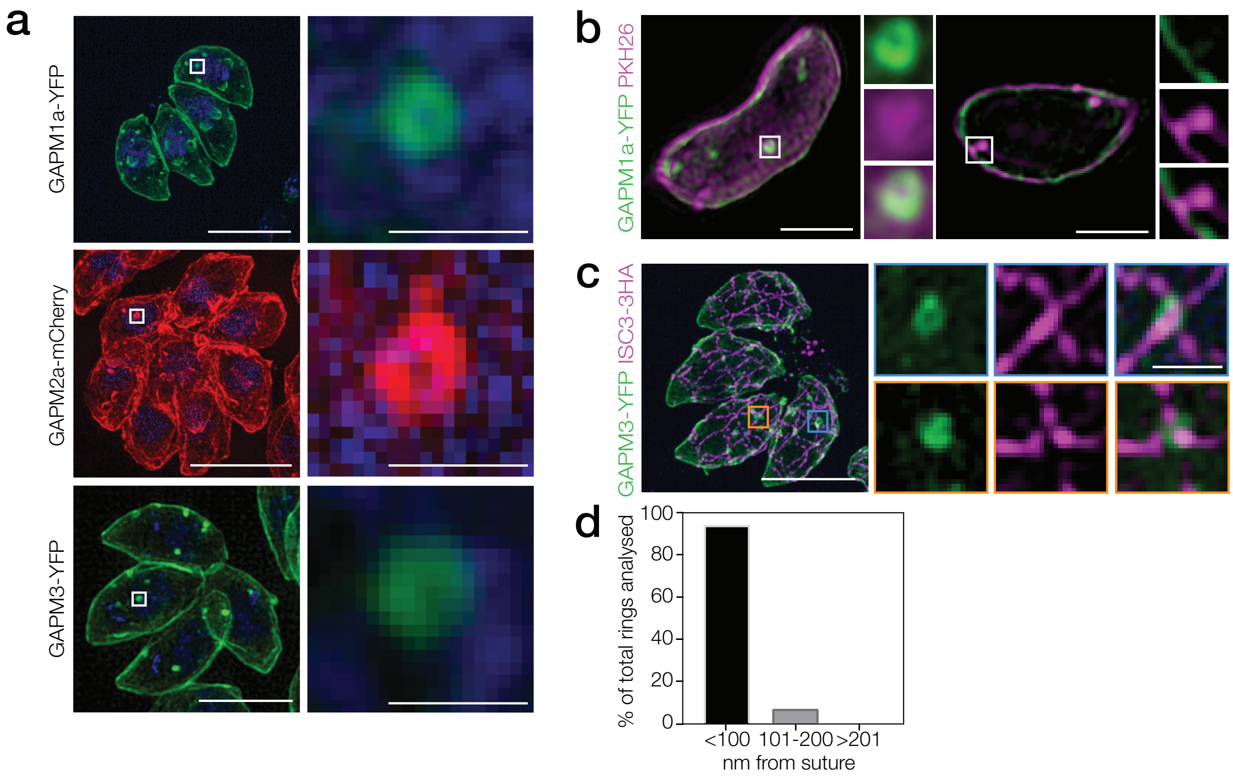

Supplementary Figure 1. GAPM foci are commonly seen at alveoli sutures. a, Imaging of GAPM1a-YFP, GAPM2a-mCherry, and GAPM3-YFP parasites using SR-SIM revealed the presence of ring structures on the IMC. Pictures are 3D reconstructions, detail scale bar 500 nm. **b**, GAPM1a-YFP expressing parasites were stained with PKH26 to visualise the plasma membrane. Clear invaginations could be seen in the plasma membrane at the site of rings. Detail scale bar 100 nm. **c**, GAPM1a-YFP rings are seen at alveoli sutures, marked by endogenous ISC3-3HA staining. Detail scale bar 500 nm. **d**, Quantification of the proportion of rings found within the indicated distances from ISC3-3HA sutures; a total of 138 rings were analysed across three independent experiments. Source data are provided as a **Source Data** file.

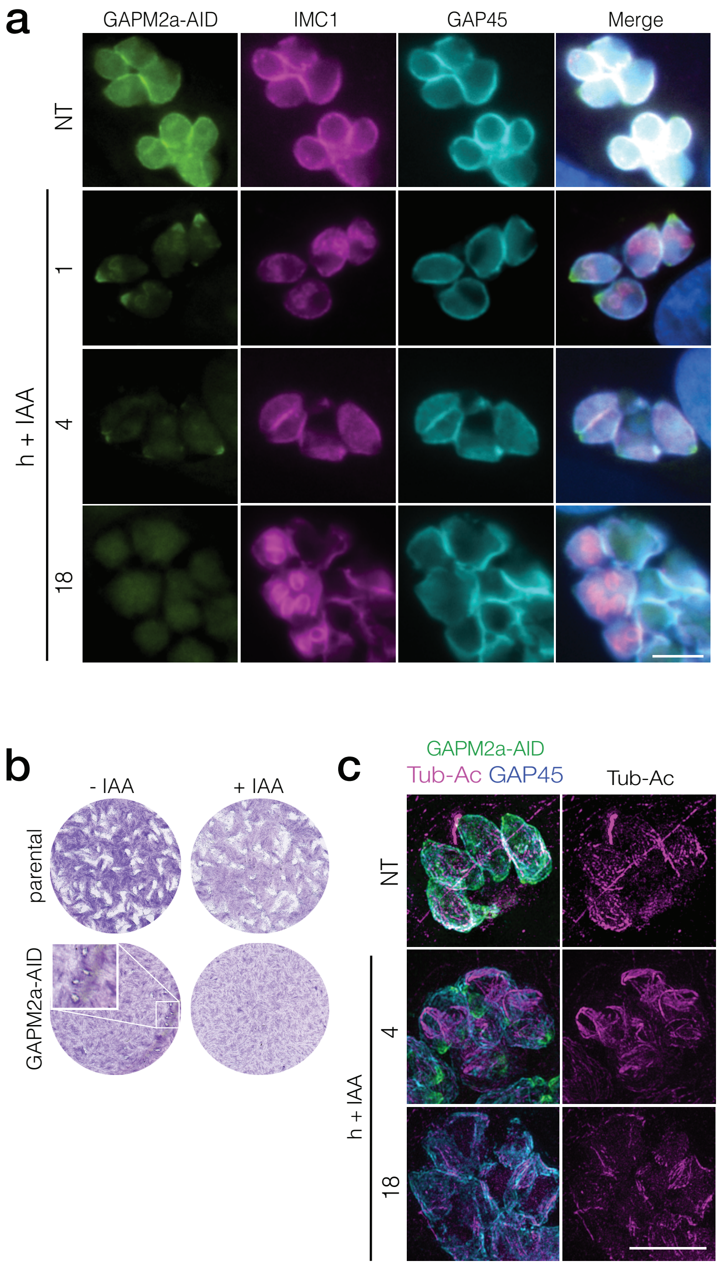

Supplementary Figure 2. Conditional depletion of GAPM2a results in a block in the lifecycle and loss of cortical microtubules. **a**, GAPM2a-AID is rapidly depleted by addition of IAA. The IMC is visualised by IMC1 (magenta) and GAP45 (cyan). Scale bar is 5 μm. **b**, Plaque assay showing that GAPM2a-AID parasites have a severe growth defect; however, no plaques are seen upon IAA addition. **c**, Cortical microtubules were visualised by anti-acetylated tubulin (magenta); IMC was delineated by GAP45 (cyan). In GAPM2a-AID parasites, microtubules lost structural organisation by 4 h post IAA addition, and after 18 h of treatment, only very few polymerised regions could be observed. Scale bar is 5 μm.

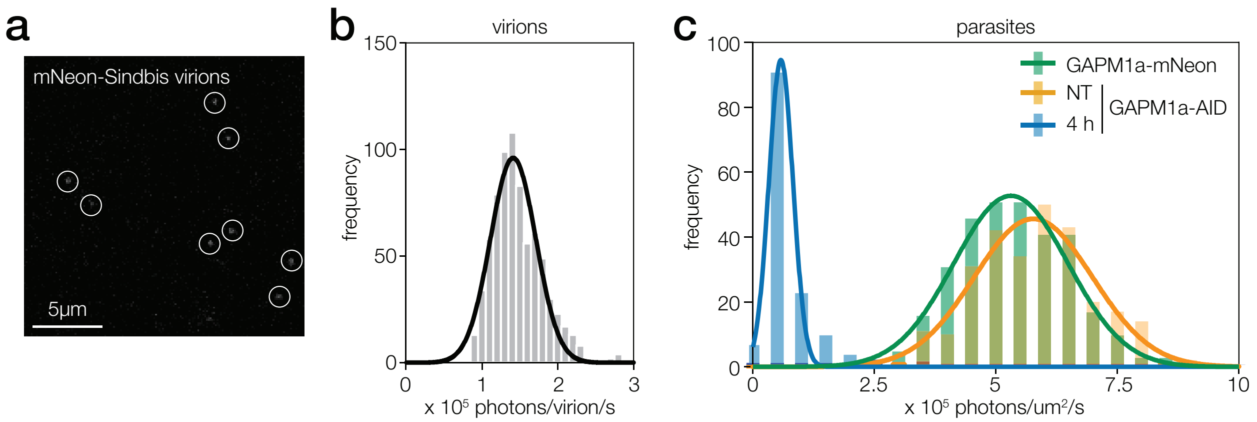

Supplementary Figure 3. Quantification of GAPM1a protein based on tagged virion standard. a, Sindbis virions incorporating mNeon-tagged TE12 was imaged; individual virions are indicated with rings. **b**, The fluorescent intensity of virions was converted to photons s^{-1} and used to generate the histogram for *n* = 915 particles. **c**, Live intracellular parasites of the strains indicated were imaged and the photons μm^{-2} s^{-1} were calculated for sections of the IMC. While the signal for the two endogenously tagged lines mostly overlapped, treatment of GAPM1a-AID parasites with IAA for 4 h resulted in a pronounced decrease in intensity. Histogram plotted for *n* > 131 parasites over two independent experiments. Source data are provided as a **Source Data** file.

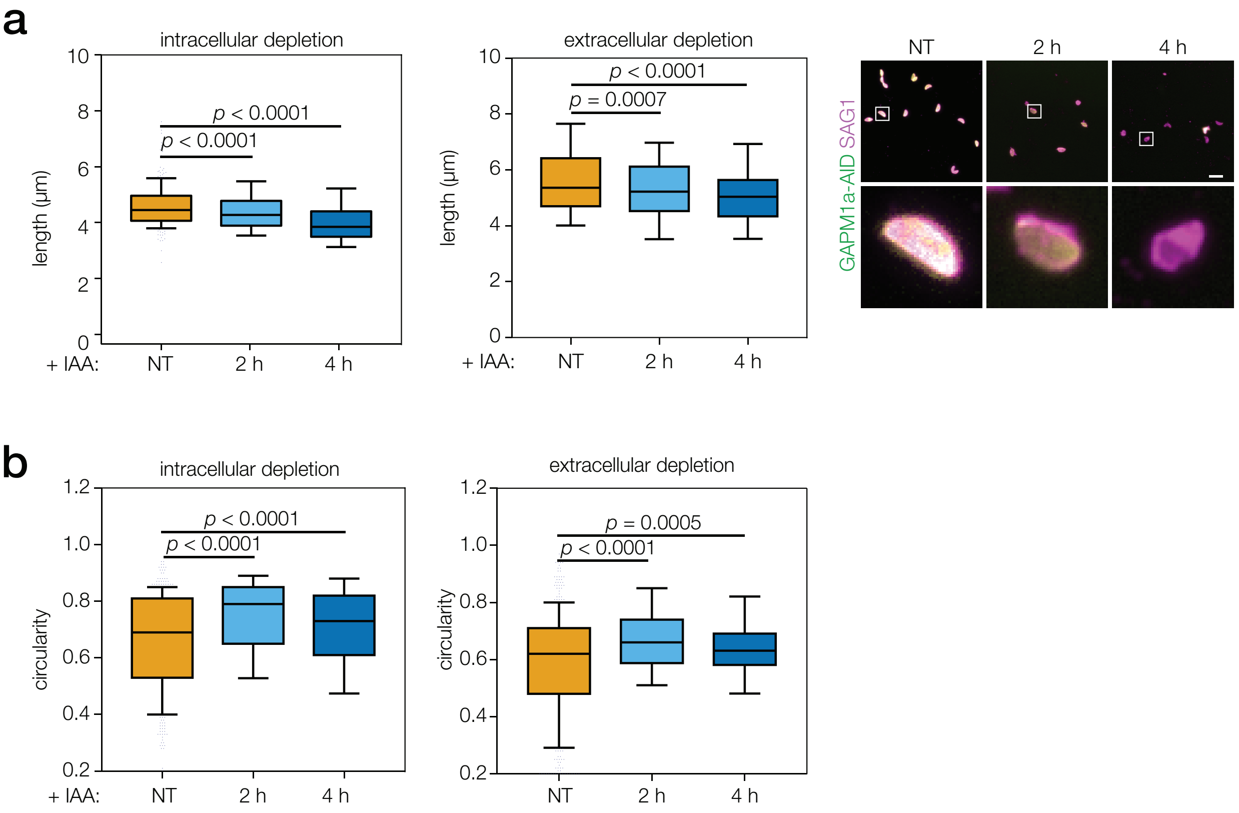

Supplementary Figure 4. Depletion of GAPM1a-AID results in a decrease in parasite length and increase in circularity. a, GAPM1a-AID parasites were treated intracellularly or extracellularly for 2 or 4 h with IAA before measuring their lengths by automated microscopy. Box plots for intracellular depletion $n = 533$ (NT), 371 (2 h), 440 (4 h) and for extracellular depletion $n = 364$ (NT), 225 (2 h), 277 (4 h) aggregated from 3 independent experiments; p values from two-tailed Student's t test. Selected images showing GAPM1a-AID (green) and SAG1 (magenta). Scale bar is 5 µm. **b**, Parasite circularity calculated for GAPM1a-AID parasites treated with IAA. Box plots for intracellular depletion $n = 403$ (NT), 223 (2 h), 363 (4 h) and for extracellular depletion $n = 420$ (NT), 282 (2 h), 299 (4h) aggregated from 3 independent experiments; p values from two-tailed Student's t test. All box plots represent median and 25th and 75th percentiles, and whiskers are at 10th and 90th percentiles. Source data are provided as a **Source Data** file.

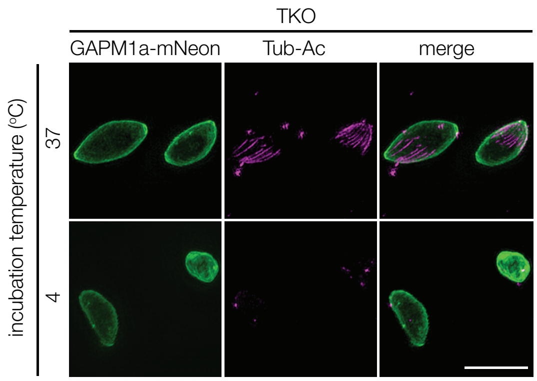

Supplementary Figure 5. Depolymerisation of microtubules does not affect GAPM1a localization. GAPM1a was endogenously tagged with mNeonGreen in the Δ*TLAP2*Δ*SPM1*Δ*TLAP3* (TKO) strain and extracellular parasites were incubated at 37 °C or 4 °C for 4 hours. Microtubules depolymerised at 4 °C; however, no change in GAPM1a-mNeon localization was observed. Scale bar is 5 µm.

SUPPLEMENTARY TABLES

Supplementary Table 1. Number of cortical microtubules and surface area across the apicomplexan zoites.

Subgroup	Species	Life cycle stage	Length (µm)	Diameter (µm)	Surface area (µm^2)	No. of MT	Reference
Coccidia	Toxoplasma gondii	Tachyzoite	6	2	68.5	22	[1]
Coccidia	Toxoplasma gondii	Microgamont	4.7	2.5	49.17	12	[2]
Coccidia	Neospora caninum	Tachyzoite	7	2	89.93	22	[3]
Coccidia	Eimeria falciformis	Sporozoite	11	6	272.22	26	[4]
Coccidia	Eimeria tenella	Sporozoite	12	3	250.15	24	[5]
Coccidia	Sarcocystis ovifelis	Sporozoite	13	7	378.12	22	[4]
Coccidia	Besnoitia jellsoni	Sporozoite	9	2	142.04	22	[4,6]
Coccidia	Cryptosporidium muris	Merozoite	8.5	1.1	119.58	10	[7]
Coccidia	Cryptosporidium muris	Sporozoite	13	1	272.68	12	[7]
Piroplasmida	Babesia bovis	Merozoite	8.5	3	139.57	32	[8]
Piroplasmida	Babesia microti	Merozoite	1.7	1.7	9.08	3	[9]
Haemosporidia	Plasmodium falciparaum	Merozite	1	1	3.14	3	[10]
Haemosporidia	Plasmodium falciparaum	Sporozoite	12	1	232.98	14	[11]
Haemosporidia	Plasmodium berghei	Sporozoite	14	1	315.54	15	[12]
Haemosporidia	Plasmodium berghei	Ookinete	10.7	2.3	199.78	32*	[13]
Haemosporidia	Plasmodium vivax	Sporozoite	12.5	1	196.42	10	[12]
Haemosporidia	Plasmodium gallinaceum	Sporozoite	12	2	243.53	11	[12]
Haemosporidia	Plasmodium gallinaceum	Ookinete	35	6	2077.81	55	[14]
Haemosporidia	Plasmodium fallax	Merozoite	1.5	3	12.07	24	[12]
Haemosporidia	Plasmodium mexicanum	Sporozoite	6	1.5	64.37	14	[15]
Haemosporidia	Plasmodium agamae	Sporozoite	6	1.8	66.78	26	[15]
Haemosporidia	Plasmodium floridense	Sporozoite	15	1	361.54	11	[16]
Haemosporidia	Haemoproteus columbae	Sporozoite	9	1	132.77	22	[17]
Haemosporidia	Leucocytozoon simondi	Ookinete	40	5	2641.73	76	[18]

Supplementary Table 1. The number of microtubules (obtained from transmission EM images) was determined from the indicated reference and compared with the estimated surface area of an ellipsoid, based on the length and diameter reported from EM images.

Supplementary Table 2. List of primers used in this study

ID	Sequence	Use
P1	TACTTCCAATCCAATTTAATGCctttcgtgaaccttacctcagc	Amplifying gapm1b 3' region for LIC cloning (F)
P2	TCCTCCACTTCCAATTTTAGCTGCTGTGCGAGAGAGGC	Amplifying gapm1b 3' region for LIC cloning (R)
P3	TACTTCCAATCCAATTTAATGCTCTACTCCGAACCGGATCGTG	Amplifying gapm2b 3' region for LIC cloning (F)
P4	TCCTCCACTTCCAATTTTAGCTAAGCTGCGCACAAGTC	Amplifying gapm2b 3' region for LIC cloning (R)
P5	TGGGGATGTCAAGTTgaggctaattagcaagcacGTTTTAGAGCTAGAA	sgRNA for C-terminal tagging of gapm1a (F)
P6	TTCTAGCTCTAAAACgtgcttgctaattagcctcAACTTGACATCCCCA	sgRNA for C-terminal tagging of gapm1a (R)
P7	TGGGGATGTCAAGTTgtgctacggtttgtgtctacGTTTTAGAGCTAGAA	sgRNA for C-terminal tagging of gapm2a (F)
P8	TTCTAGCTCTAAAACgtagacacaaaccgtagcacAACTTGACATCCCCA	sgRNA for C-terminal tagging of gapm2a (R)
P9	gctgctgcggagcaggctcaggcttgcctgtcctgcagatttatggtgagcaagggcgagg agg	Amplifying mNeon-AID for gapm1a tagging (F)
P10	gcacggcctccagttactgtcgcttctcctgttcaccacatttcccagTTAATCGAGCG GGTCCTGGTTC	Amplifying mNeon-AID for gapm1a tagging (R)
P11	cgaggtcgaaatgggtgttgtgaaccccaactaccagtccatggtgagcaagggcgagga gg	Amplifying mNeon-AID for gapm2a tagging (F)
P12	atcccccatccaggttacccgaaaaacgcgcatttctgtcTTAATCGAGCGGGTCC TGGTTC	Amplifying mNeon-AID for gapm2a tagging (R)
P13	ggcacCCTAGGATGGCGCAGGTTCAGCTGG	Amplifying GFPnanobody (F)
P14	gaGCCAGGGGCCGAGACGGCCGGTCAGTCACGATGCGGCCGCT	Amplifying GFPnanobody (R)
P15	ggtaaGAATTCATGGTGAGCAAGGGCGAGGA	Amplifying mCherry to insert into dd-Myc-GFPnanobody (F)
P16	cggccGAATTCCTTGTACAGCTCGTCCA	Amplifying mCherry to insert into dd-Myc-GFPnanobody (R)
P17	cgcgaggtcgaaatgggtgttgtgaaccccaactaccagtccATGGTGAGCAAGG GCGAGGAGG	Amplifying mCherry for gapm2a tagging (F)
P18	atcccccatccaggttacccgaaaaacgcgcatttctgtcTTACTTGTACAGCTCGT CCA	Amplifying mCherry for gapm2a tagging (R)
P19	catggtcatgggtggtatgaagtctcagacttccatgctgATGGTGAGCAAGGGCG AGGAGG	Amplifying mCherry for gapm3 tagging (F)
P20	gttctgtacacggcaatcatcacctgtgtctaagacgaacTTACTTGTACAGCTCGT CCA	Amplifying mCherry for gapm3 tagging (R)
P21	ggATCCACTAGTTctagaggtacCGTTTGAAATTCAGGTGACAGATGC	Amplify ATPase synthase beta 5'UTR (F)
P22	CCATGGTGGCgctagcTTTCGCAAAGGTTTGCCGTAG	Amplify ATPase synthase beta 5'UTR (R)
P23	CTTTGCGAAAgctagcGCCACCATGGAGCAGAAGCTGATTTCTGAG GAAGATCTGGGCAC	Amplify GFP-OMP (F)
P24	cagcttctgtcctaggTCAGAGCTGCTTTCGGTATCTCACGAAGGCCCAA ACTGC	Amplify GFP-OMP (R)

SUPLEMENTARY REFERENCES

1 Dubey, J. P., Lindsay, D. S. & Speer, C. A. Structures of Toxoplasma gondii tachyzoites, bradyzoites, and sporozoites and biology and development of tissue cysts. *Clin Microbiol Rev* **11**, 267-299 (1998).

2 Speer, C. A. & Dubey, J. P. Ultrastructural differentiation of Toxoplasma gondii schizonts (types B to E) and gamonts in the intestines of cats fed bradyzoites. *Int J Parasitol* **35**, 193-206, doi:10.1016/j.ijpara.2004.11.005 (2005).

3 Lindsay, D. S., Speer, C. A., Toivio-Kinnucan, M. A., Dubey, J. P. & Blagburn, B. L. Use of infected cultured cells to compare ultrastructural features of Neospora caninum from dogs and Toxoplasma gondii. *Am J Vet Res* **54**, 103-106 (1993).

4 D'Haese, J., Mehlhorn, H. & Peters, W. Comparative electron microscope study of pellicular structures in coccidia (Sarcocystis, Besnoitia and Eimeria). *Int J Parasitol* **7**, 505-518 (1977).

5 Ryley, J. F. Ultrastructural Studies on Sporozoite of Eimeria Tenella. *Parasitology* **59**, 67-+, doi:Doi 10.1017/S0031182000069833 (1969).

6 Ayroud, M., Leighton, F. A. & Tessaro, S. V. The morphology and pathology of Besnoitia sp. in reindeer (Rangifer tarandus tarandus). *J Wildl Dis* **31**, 319-326, doi:10.7589/0090-3558-31.3.319 (1995).

7 Uni, S., Iseki, M., Maekawa, T., Moriya, K. & Takada, S. Ultrastructure of Cryptosporidium muris (strain RN 66) parasitizing the murine stomach. *Parasitol Res* **74**, 123-132 (1987).

8 Potgieter, F. T., Els, H. J. & Vuuren, A. S. The fine structure of merozoites of Babesia bovis in the gut epithelium of Boophilus microplus. *Onderstepoort J Vet Res* **43**, 1-9 (1976).

9 Rudzinska, M. A., Spielman, A., Riek, R. F., Lewengrub, S. J. & Piesman, J. Intra-Erythrocytic Gametocytes of Babesia-Microti and Their Maturation in Ticks. *Canadian Journal of Zoology-Revue Canadienne De Zoologie* **57**, 424-434, doi:DOI 10.1139/z79-050 (1979).

10 Morrissette, N. S. & Sibley, L. D. Cytoskeleton of apicomplexan parasites. *Microbiol Mol Biol Rev* **66**, 21-38; table of contents (2002).

11 Russell, D. G. & Burns, R. G. The polar ring of coccidian sporozoites: a unique microtubule-organizing centre. *J Cell Sci* **65**, 193-207 (1984).

12 Aikawa, M. Parasitological review. Plasmodium: the fine structure of malarial parasites. *Exp Parasitol* **30**, 284-320 (1971).

13 Guttery, D. S. *et al.* A Unique Protein Phosphatase with Kelch-Like Domains (PPKL) in Plasmodium Modulates Ookinete Differentiation, Motility and Invasion. *Plos Pathogens* **8**, doi:ARTN e1002948 10.1371/journal.ppat.1002948 (2012).

14 Raibaud, A. *et al.* Cryofracture electron microscopy of the ookinete pellicle of Plasmodium gallinaceum reveals the existence of novel pores in the alveolar membranes. *J Struct Biol* **135**, 47-57, doi:10.1006/jsbi.2001.4396 (2001).

15 Telford, S. *Hemoparasites of the Reptilia : color atlas and text.* (Taylor & Francis, 2008).

16 Klein, T. A., Akin, D. C., Young, D. G. & Telford, S. R., Jr. Sporogony, development and ultrastructure of Plasmodium floridense in Culex erraticus. *Int J Parasitol* **18**, 711-719 (1988).

17 Klei, T. R. The fine structure of Haemoproteus columbae sporozoites. *J Protozool* **19**, 281-286 (1972).

18 Brockley Paterson, W. & Desser, S. S. The polar ring complex in ookinetes of Leucocytozoon simondi (Apicomplexa: Haemosporina) and evidence for a conoid in haemosporidian ookinetes. *Eur J Protistol* **24**, 244-251, doi:10.1016/S0932-4739(89)80061-6 (1989).
