# Supplementary material for: Alveolar proteins stabilize cortical microtubules in Toxoplasma gondii
Source: Nat Commun. 2019 Jan 23;10:401. doi: 10.1038/s41467-019-08318-7 (PMC6344517; doi:10.1038/s41467-019-08318-7)
Supplement: Supplementary file 3 — Description of Additional Supplementary Files [file 41467_2019_8318_MOESM3_ESM.pdf]

Description of Additional Supplementary Files

File Name: Supplementary Movie 1
Description: GAPM1a-YFP parasites were imaged every 7 minutes by live 3DSIM. Stacks were projected and representative frames shown in Fig. 1e. Representative of three videos from two independent experiments. Scale bar is 5 µm

File Name: Supplementary Movie 2
Description: Untreated GAPM1a-AID on poly-L coated coverslips were allowed to glide and imaged continuously for 1 min. Scale bar is 5 µm.

File Name: Supplementary Movie 3
Description: GAPM1a-AID parasites treated intracellularly for 4 h were placed on poly-L coated coverslips and allowed to glide. Scale bar is 5 µm

File Name: Supplementary Movie 4
Description: Video of host cells infected with wild-type parasites and treated with zaprinast to trigger egress. Representative frames shown in Fig. 3d, top panel.

File Name: Supplementary Movie 5
Description: Video of host cells infected with GAPM1-AID parasites and treated with zaprinast to trigger egress. Representative frames shown in Fig. 3d, middle panel.

File Name: Supplementary Movie 6
Description: Video of host cells infected with GAPM1-AID parasites, treated for 18 h with IAA and treated with zaprinast to trigger egress. Representative frames shown in Fig. 3d, bottom panel.

File Name: Supplementary Movie 7
Description: Untreated GAPM1a-AID/mTagRFP-T_TubA1 parasites were imaged every 7 mins for around 190 minutes. Representative frames shown in Fig. 5b, top panel. Representative of 12 videos from two independent experiments.

File Name: Supplementary Movie 8
Description: IAA was added to GAPM1a-AID/ mTagRFP-T_TubA1 parasites and imaging started immediately. Frames were taken every 7 mins for around 190 minutes. Representative frames shown in Fig. 5b, lower panel. Representative of 12 videos from two independent experiments.

File Name: Supplementary Movie 9
Description: Untreated GAPM1a-AID/ mTagRFP-T_TubA1 parasites imaged every 7 mins for around 150 minutes. Representative frames shown in Fig. 5a, upper panel. Scale bar is 5 µm.

File Name: Supplementary Movie 10
Description: IAA was added to GAPM1a-AID/ mTagRFP-T_TubA1 parasites and imaging started immediately. Frames were taken every 7 mins for around 150 minutes. Representative frames shown in Fig. 5a, lower panel. Scale bar 5 µm.
