# Supplementary material for: Alveolar proteins stabilize cortical microtubules in Toxoplasma gondii
Source: Nat Commun. 2019 Jan 23;10:401. doi: 10.1038/s41467-019-08318-7 (PMC6344517; doi:10.1038/s41467-019-08318-7)
Supplement: Supplementary file 15 — Reporting Summary [file 41467_2019_8318_MOESM15_ESM.pdf]

nature research

Corresponding author(s): Clare Harding
Sebastian Lourido

Last updated by author(s): 2018-21-12

Reporting Summary

Nature Research wishes to improve the reproducibility of the work that we publish. This form provides structure for consistency and transparency in reporting. For further information on Nature Research policies, see Authors & Referees and the Editorial Policy Checklist.

Statistics

For all statistical analyses, confirm that the following items are present in the figure legend, table legend, main text, or Methods section.

n/a	Confirmed	
☐	☒	The exact sample size (n) for each experimental group/condition, given as a discrete number and unit of measurement
☐	☒	A statement on whether measurements were taken from distinct samples or whether the same sample was measured repeatedly
☐	☒	The statistical test(s) used AND whether they are one- or two-sided<br>*Only common tests should be described solely by name; describe more complex techniques in the Methods section.*
☒	☐	A description of all covariates tested
☐	☒	A description of any assumptions or corrections, such as tests of normality and adjustment for multiple comparisons
☐	☒	A full description of the statistical parameters including central tendency (e.g. means) or other basic estimates (e.g. regression coefficient) AND variation (e.g. standard deviation) or associated estimates of uncertainty (e.g. confidence intervals)
☒	☐	For null hypothesis testing, the test statistic (e.g. F, t, r) with confidence intervals, effect sizes, degrees of freedom and P value noted<br>*Give P values as exact values whenever suitable.*
☒	☐	For Bayesian analysis, information on the choice of priors and Markov chain Monte Carlo settings
☒	☐	For hierarchical and complex designs, identification of the appropriate level for tests and full reporting of outcomes
☒	☐	Estimates of effect sizes (e.g. Cohen's d, Pearson's r), indicating how they were calculated

Our web collection on statistics for biologists contains articles on many of the points above.

Software and code

Policy information about availability of computer code

Data collection	Data was collected manually or using ImageJ macros.
Data analysis	Data was analyzed using Microsoft Excel, GraphPad Prism and BitPlane Imaris.

For manuscripts utilizing custom algorithms or software that are central to the research but not yet described in published literature, software must be made available to editors/reviewers. We strongly encourage code deposition in a community repository (e.g. GitHub). See the Nature Research guidelines for submitting code & software for further information.

Data

Policy information about availability of data

All manuscripts must include a data availability statement. This statement should provide the following information, where applicable:

- Accession codes, unique identifiers, or web links for publicly available datasets
- A list of figures that have associated raw data
- A description of any restrictions on data availability

The source data underlying Figures 2g, 3a–c, 4c, 4f, 4h, 5a–e, and 5g, and Supplementary Figures 1d, 3a–c, and 4a–b are provided as a Source Data file. All other data is available from the corresponding authors by request.

Field-specific reporting

Please select the one below that is the best fit for your research. If you are not sure, read the appropriate sections before making your selection.

☒ Life sciences ☐ Behavioural & social sciences ☐ Ecological, evolutionary & environmental sciences

Life sciences study design

All studies must disclose on these points even when the disclosure is negative.

Sample size	Multiple biological replicates were used in all experiments. Exact sample size is provided in each figure legend.
Data exclusions	No data was excluded from the analyses.
Replication	Experiments were performed with multiple biological replicates on different days. See methods for details.
Randomization	Assignment of strains to treatment groups was randomized between biological replicates.
Blinding	Investigators were not blinded to group assignments.

Reporting for specific materials, systems and methods

We require information from authors about some types of materials, experimental systems and methods used in many studies. Here, indicate whether each material, system or method listed is relevant to your study. If you are not sure if a list item applies to your research, read the appropriate section before selecting a response.

Materials & experimental systems

n/a	Involved in the study	
☐	☒	Antibodies
☐	☒	Eukaryotic cell lines
☒	☐	Palaeontology
☒	☐	Animals and other organisms
☒	☐	Human research participants
☒	☐	Clinical data

Methods

n/a	Involved in the study	
☒	☐	ChIP-seq
☒	☐	Flow cytometry
☒	☐	MRI-based neuroimaging

Antibodies

Antibodies used	Information is included for all commercial antibodies used. For non-commercial antibodies the source is listed along with the dilution factors used.
Validation	All antibodies had been validated commercially, or as part of previously published studies.

Eukaryotic cell lines

Policy information about cell lines

Cell line source(s)	Primary human foreskin fibroblasts (HFFs) were obtained from ATCC (ATCC SCRC-1041). Published Toxoplasma cell lines were obtained from colleagues and used to generate additional strains following the materials and methods. Generated cell lines are available upon request from the corresponding authors.
Authentication	HFFs were authenticated by the ATCC. Toxoplasma strains were genotyped by PCR and sequencing, as appropriate.
Mycoplasma contamination	HFF human cells were frequently tested and found negative for Mycoplasma. Parasite lines were not tested.
Commonly misidentified lines (See ICLAC register)	n/a
